# Retraction: Severe Dengue With Multisystem Inflammatory Syndrome in Children Due to COVID-19: A Co-infection Case Series

**DOI:** 10.7759/cureus.r35

**Published:** 2021-11-16

**Authors:** Anima Ferdous, M Monir Hossain, Manifa Afrin

**Affiliations:** 1 Pediatric Intensive Care Unit, Universal Medical College Hospital, Dhaka, BGD; 2 Pediatric Medicine, Universal Medical College Hospital, Dhaka, BGD

This article has been retracted due to numerous errors and incorrect conclusions. Certain criteria is required in order to identify MIS-C, including no other attributable cause for the fever. All patients presented with acute dengue fever, thus the clinical condition cannot be called MIS-C. Also, while Covid-19 antibodies were positive, this could be the result of prior infection. Therefore there is no proof of the patients having acute Covid-19, thus invalidating the conclusions. We deeply regret that these issues were not identified prior to publication.

